# The Effect of Sugammadex Versus Neostigmine on Postoperative Cognitive Recovery: A Systematic Review and Meta-Analysis

**DOI:** 10.7759/cureus.109071

**Published:** 2026-05-18

**Authors:** Hudson Martins de Brito, Daniel Ferraz Pozzer Gularte, Vitória de Queiroz Vasconcelos, Francisco Willamy Pedrosa Alves Filho, Nágila Alves Lima, Alecia Lynn Sabartinelli Stein, Tiago de Almeida Macruz

**Affiliations:** 1 Department of Medicine, Federal University of Ceará, Fortaleza, BRA; 2 Department of Surgery, Souza Marques University, Rio de Janeiro, BRA; 3 Department of Radiology, Cancer Institute of Ceará, Fortaleza, BRA; 4 Department of Surgery, Federal University of Ceará, Sobral, BRA; 5 Department of Surgery, Christus University Center, Fortaleza, BRA; 6 Department of Anesthesiology, University of Miami, Miami, USA

**Keywords:** anesthesia recovery period, mental cognition, neostigmine, neuromuscular blocking agents, postoperative complications, sugammadex

## Abstract

Postoperative cognitive dysfunction is a major clinical concern, particularly in elderly patients, leading to reduced quality of life and increased mortality. The impact of neuromuscular blockade reversal agents, such as sugammadex and neostigmine, on cognitive recovery remains uncertain.

A systematic search of PubMed, EMBASE, and Cochrane databases identified randomized controlled trials (RCTs) and observational studies comparing sugammadex and neostigmine (± anticholinergics) for reversal of neuromuscular blockade in adults under general anesthesia for elective surgery. Studies published up to February 2025 that used the Mini-Mental State Examination (MMSE) or the cognitive domain of the Postoperative Quality Recovery Scale (PQRS) were included, as they both evaluate cognition on multiple aspects. Standardized mean differences (SMD) and standard errors (SE) were calculated, and a meta-analysis using the generic inverse variance method was performed. Heterogeneity was assessed with the I² statistic.

Six RCTs and three observational studies, including 1,116 patients, were analyzed. Sugammadex was associated with better early cognitive recovery (40 to 60 minutes postoperatively) compared to neostigmine (SMD 0.20; 95% CI (0.07, 0.33); p = 0.003). No significant cognitive differences were observed immediately (≤15 minutes), on day one, or on days three to seven postoperatively. Sugammadex also improved physiological recovery at 40 minutes (RR 1.20; 95% CI (1.09, 1.30); p < 0.0001). Sensitivity analysis confirmed these findings. No differences were found in nociceptive, emotive, activities of daily living, or overall recovery domains.

Sugammadex may improve early postoperative cognitive and physiological recovery over neostigmine, which could lead to a faster post-anesthesia care unit (PACU) discharge. No differences were observed at other time points or PQRS domains.

## Introduction and background

Postoperative cognitive dysfunction (POCD) refers to a measurable decline in cognition following surgery compared to preoperative function [1]. It is important to emphasize that POCD is currently considered part of the broader spectrum of postoperative neurocognitive disorders (PNCD) [1].

Clinically, POCD is characterized by the emergence of new cognitive impairments post-surgery, including memory deficits, challenges in multitasking, and diminished psychomotor coordination [2]. These impairments are typically subtle and may persist beyond the immediate postoperative period [1]. It is associated with short- and long-term complications, mainly in elderly patients, including a decrease in quality of life [3], delirium [4], increased mortality, risk of leaving the labor market prematurely, social dependency [5], and potentially leading to persistent cognitive decline and dementia [6,7]. It is particularly prevalent in cardiac and major orthopedic surgeries [3,5]. The exact etiology of POCD remains unclear, but current evidence suggests that the cholinergic system plays a significant role in its development [8,9], along with other contributing factors such as neuroinflammation, oxidative stress, and genetic susceptibility [10].

Another important condition within the spectrum of PNCD is postoperative delirium (POD), which is characterized by acute disturbances in attention, awareness, and conditions following surgical procedures. Postoperative delirium typically develops within hours to days after surgery, presents with a fluctuating course, and tends to improve with appropriate management during the postoperative period [4]. Clinically, it can be classified into hyperactive, hypoactive, and mixed [4]. Moreover, the diagnosis of PNCD imposes a considerable economic burden on healthcare systems, driven by increased costs [11]. In this context, another important point to emphasize is that cognitive recovery is the process by which the patients return to their preoperative cognitive baseline within the immediate hours to days following surgery [12].

The choice of neuromuscular blockade reversal agent may impact postoperative cognitive recovery. Cholinesterase inhibitors such as neostigmine increase acetylcholine levels at the neuromuscular junction by inhibiting acetylcholinesterase. Although traditionally believed not to cross the blood-brain barrier (BBB), emerging evidence suggests that the BBB is dynamically regulated and may become more permeable due to anesthesia and surgical stress through neuroinflammation [13,14]. This increased permeability could allow these agents to enter the central nervous system and alter cholinergic signaling. Additionally, anticholinergic agents like atropine or glycopyrrolate are often co-administered to mitigate peripheral muscarinic side effects [15]. Some of these, particularly atropine, readily cross the BBB and may further disrupt central cholinergic function, potentially contributing to postoperative cognitive dysfunction [16].

Sugammadex offers an alternative approach. It is a modified gamma-cyclodextrin that selectively binds to aminosteroid neuromuscular blockers like rocuronium and vecuronium, forming a complex that is excreted by the kidneys [17]. This mechanism reduces the concentration of free neuromuscular blockers at the motor end plate, providing rapid and specific reversal of neuromuscular blockade [18] without directly affecting the cholinergic system. In light of this, we aim to test the hypothesis that patients receiving sugammadex for neuromuscular blockade reversal exhibit better cognitive recovery in the postoperative period compared to those receiving neostigmine.

## Review

Methods

Protocol and Registration

The systematic review and meta-analysis were conducted following the guidelines set forth by the Cochrane Collaboration [19] and presented as recommended by the Preferred Reporting Items for Systematic Reviews and Meta-Analyses (PRISMA) statement [20]. The study protocol was registered in PROSPERO (the International Prospective Register of Systematic Reviews) under the code CRD420251002188.

Information Sources

A systematic search was conducted in PubMed, EMBASE, and the Cochrane databases. Studies published up to February 2025 were considered. The search was initially performed on February 26th, 2025, and updated on September 24th, 2025, to include more recent studies. Gray literature was not searched.

Search Strategy

The search strategy combined terms related to sugammadex, neostigmine, anticholinergic agents, and cognitive outcomes. The search strategy was the following: (sugammadex OR Bridion OR “selective relaxant binding agent” OR “selective relaxant binding agents” OR SRBA) AND (neostigmine OR atropine OR anticholinergic OR anticholinergics OR anti-cholinergic OR anti-cholinergics OR acetylcholinesterase OR cholinesterase OR cholinergic OR cholinergics OR muscarinic OR muscarinics OR antimuscarinic OR anti-muscarinic OR antimuscarinics OR anti-muscarinics OR glycopyrronium OR glycopyrrolate) AND (delirium OR “cognitive dysfunction” OR cognition OR cognitive OR agitation OR confusion OR orientation OR disorientation OR delusion OR delusions OR hallucination OR hallucinations OR psychosis) (Table 1).

**Table 1:** Literature search strategy

Database	Search terms
PubMed	(sugammadex OR Bridion OR “selective relaxant binding agent” OR “selective relaxant binding agents” OR SRBA) AND (neostigmine OR atropine OR anticholinergic OR anticholinergics OR anti-cholinergic OR anti-cholinergics OR acetylcholinesterase OR cholinesterase OR cholinergic OR cholinergics OR muscarinic OR muscarinics OR antimuscarinic OR anti-muscarinic OR antimuscarinics OR anti-muscarinics OR glycopyrronium OR glycopyrrolate) AND (delirium OR “cognitive dysfunction” OR cognition OR cognitive OR agitation OR confusion OR orientation OR disorientation OR delusion OR delusions OR hallucination OR hallucinations OR psychosis).
EMBASE	Same as PubMed
Cochrane	Same as PubMed

Eligibility Criteria

Studies were included if they were randomized controlled trials (RCTs) or observational studies comparing sugammadex and neostigmine, with or without anticholinergics (atropine or glycopyrrolate), for neuromuscular blockade reversal in adult patients (≥18 years) undergoing elective surgery under general anesthesia. Studies assessing cognitive recovery as an outcome using either the Mini-Mental State Examination (MMSE) or the cognitive domain of the Postoperative Quality Recovery Scale (PQRS) were included. Studies were excluded if they involved patients under 18 years of age, those classified as American Society of Anesthesiologists (ASA) IV, or those undergoing non-elective surgery (Table 2). Observational studies were included to broaden the evidence base and enhance the generalizability of findings.

**Table 2:** Inclusion and exclusion criteria of population in the search RCTs: Randomized controlled trials, ASA: American Society of Anesthesiologists

Inclusion criteria	Exclusion criteria
RCTs and observational studies, including prospective or retrospective cohorts	Case reports, case series, reviews, editorials, letters, and conference abstracts
Studies including predominantly ASA I–III patients were prioritized	Patients classified as ASA IV
Studies evaluating neuromuscular blockade reversal using sugammadex or neostigmine	Studies in animals or in vitro, lacking sufficient data extraction, duplicate publications, or studies with overlapping populations
Adult patients (≥ 18 years) undergoing elective surgery under general anesthesia	Patients <18 years of age, or undergoing emergency surgery

Outcomes

The primary outcome was postoperative cognitive recovery, defined as the process of returning to preoperative cognitive function in the postoperative setting, in different time frames, using the aforementioned scales. Secondary outcomes included other key dimensions of the PQRS: physiological, nociceptive, emotive, and activities of daily living (ADL) domains, along with an assessment of overall recovery.

Study Selection and Data Extraction

Two authors independently screened studies for eligibility, with discrepancies resolved through consultation with a third author. Data extraction was performed using a shared table, where two authors independently reviewed and extracted key data, including study design, sample size, patient characteristics, interventions, comparators, and outcomes. For data presented only in graphical form, WebPlotDigitizer Software version 5.2 (Ankit Rohatgi, Automeris LLC, Plantation, FL, USA) was used for extraction.

Risk of Bias Assessment

Risk of bias was independently assessed by two investigators using the Cochrane Risk of Bias tool (RoB2) for RCTs [21] and the risk of bias in non-randomised studies of interventions (ROBINS-I) tool for observational studies [22]. Discrepancies were resolved through discussion, and if consensus was not reached, a third investigator was consulted.

Data Standardization

To address the variability in the timing of cognitive assessments across studies, we standardized the evaluation intervals into four distinct periods: immediate postoperative (within 15 minutes in the postoperative room), early postoperative (between 40 and 60 minutes), intermediate (one day after surgery), and late (between postoperative days three and seven). Although the MMSE and the cognitive domain of the PQRS differ in structure, both evaluate multiple domains within a global framework.

However, the MMSE was not originally designed for immediate postoperative use. Consequently, findings from this tool during the immediate and early periods should be interpreted with caution, as residual anesthetic effects may influence performance [12,23,24]. In this review, cognitive recovery was defined as the degree to which patients return to their preoperative cognitive baseline, based on comparisons between pre and postoperative scores. This distinction is essential, as the concept of POCD refers to persistent cognitive decline over longer follow-up periods (typically three to 12 months), which was beyond the scope of the present analysis. 

Effect Measures

In line with the Cochrane Handbook for Systematic Reviews of Interventions [19], we therefore combined their results using standardized mean differences (SMD) and standard errors (SE) to express outcomes on a common scale. Standardization was conducted using the Practical Meta-Analysis Effect Size Calculator version 2023.11.27 (Wilson DB, Campbell Corporation, Philadelphia, PA, USA) [25]. This approach unified cognitive assessment tools and converted postoperative scores to a unit-free metric, resolving inconsistencies in POCD definitions. Rather than relying on variable diagnostic thresholds (e.g., declines of one or two standard deviations), we focused on standardized quantitative outcomes to minimize study-specific assumptions and ensure objective, comparable measures of cognitive recovery.

Statistical Analysis and Sensitivity Analysis

Meta-analysis was conducted using a random-effects model and the generic inverse variance method in RevMan (Version 5.4; Cochrane Collaboration, 2023) to estimate pooled effect sizes with 95% CI. For secondary outcomes, which consisted exclusively of dichotomous data, risk ratios (RR) were calculated, and pooled data were analyzed using the Mantel-Haenszel (M-H) test under a random-effects model [26]. Statistical analyses were conducted only if data from at least four studies were available for each time frame or each domain of the PQRS at the specified time points. Meta-regression and subgroup analyses were preplanned to explore potential sources of heterogeneity, including benzodiazepine use, cognitive assessment tool, anticholinergic exposure, surgical type, and patient age.

For studies reporting continuous outcomes as means and ranges, standard deviations were estimated using the d2-based method described by Hozo et al. (2005) [27], and for those reporting them as medians and interquartile range, means and standard deviations were estimated using the method described by Abbas et al. (2024) [28]. When only means and 95% confidence intervals were provided, standard deviations were imputed using the method described by Luo et al. (2018) [29].

Bias and Certainty Assessment

The potential for small study bias was assessed through visual inspection of contour-enhanced funnel plots. Sensitivity analyses were conducted on the primary outcome using the leave-one-out method. Heterogeneity was evaluated using the I² statistic, with statistical significance defined as a p-value < 0.05.

Results

Study Selection and Baseline Characteristics

**Figure 1:**
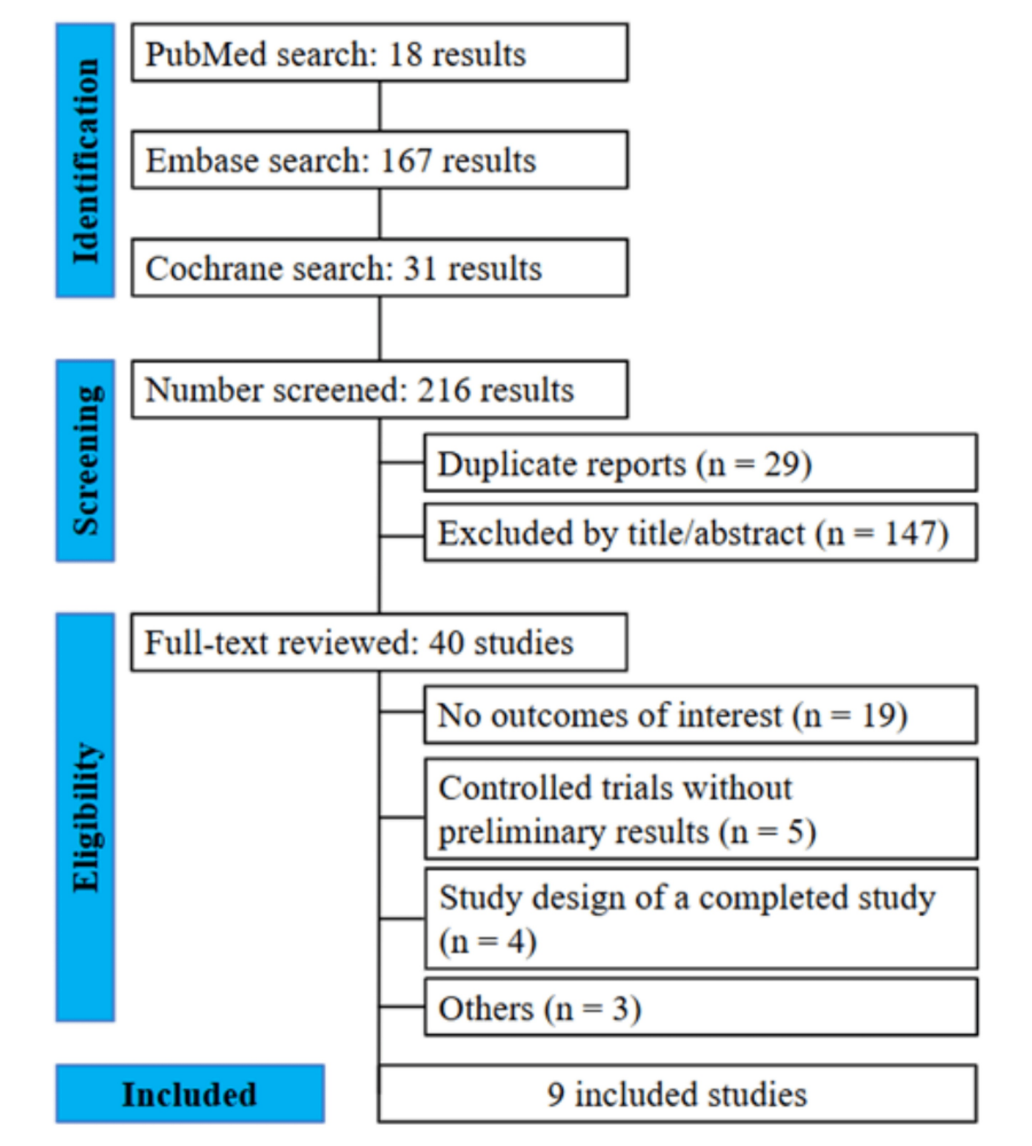
PRISMA flow diagram of study screening and selection PRISMA: Preferred Reporting Items for Systematic Reviews and Meta-Analyses [20]

As detailed in Figure 1, application of the search strategy resulted in 216 results. After removal of duplicates and exclusion of studies based on title or abstract, 40 studies were fully reviewed for inclusion and exclusion criteria. Conclusively, nine studies, including six RCTs and three observational studies, were included in the analysis, featuring a total of 1,116 patients. Sugammadex was used for neuromuscular blockade reversal in 557 patients, and 559 received neostigmine (± anticholinergics).

Regarding the category 'others' for study exclusion, two of the three studies were excluded for not assessing cognition using the PQRS scale, whereas the remaining study was excluded due to the absence of a clearly defined timeline for PQRS assessment. Characteristics of each study are presented in Table 3 [30-38]. 

**Table 3:** Baseline characteristics of included studies ¹Data expressed in mean or median. *Anesthesia time was estimated from surgery time in studies that did not specify it. SG: Sugammadex; NG: Nugammadex; N/S: Not standardized; CDT: Clock-drawing test; IST: Isaacs Set Test; MMSE: Mini‐Mental State Examination; MoCA: Montreal Cognitive Assessment; PQRS: Postoperative Quality Recovery Score; RCT: Randomized controlled trial

Study	Design	N	Male (%)	Age¹	BMI¹	Type of surgery	Intervention	Control	Anesthetic induction	Anesthetic maintenance	Neuromuscular induction blocker	Anesthesia time (min)¹* SG/NG	Cognitive evaluation scale
Amorim et al., 2014 [30]	Cohort	53/48	49/33	42/50	25/27	Multiple	Sugammadex not standadized (N/S) dose	Neostigmine N/S dose	N/S	N/S	N/S	100/79	PQRS
Batistaki et al., 2017 [31]	RCT	78/82	36/46	62/61	28/28	Multiple	Sugammadex 2 mg/kg	Neostigmine 0.04 mg/kg + atropine 0.4mg/mg of Ng	Propofol 2-2.5 mg/kg + fentanyl 2 μg/kg	Sevoflurane	Rocuronium 0.8 mg/kg	129/113	MMSE/clock-drawing test (CDT)/Isaacs set test (IST)
Boggett et al., 2020 [32]	RCT	175/175	55/72	54/55	29/29	Multiple	Sugammadex 2-4 mg/kg	Neostigmine 0.15 mg/kg + atropine 20 μg/kg or glycopyrrolate 5 μg/kg	Propofol + Fentanyl 0.5-3 μg/kg or remifentanil	Desflurane	Rocuronium 0.6-1.2 mg/kg	164/165	PQRS
Claroni et al., 2019 [33]	RCT	54/55	78/73	63/60	26/26	Robotic-assisted radical cystectomy	Sugammadex 2 mg/kg	Neostigmine 0.04 mg/kg + atropine 0.02 mg/kg	Propofol 2 mg/kg + fentanyl 3-5 mg/kg	Sevoflurane + remifentanil 2-4 ng/mL	Rocuronium 0.7 mg/kg	378/361	MMSE
Kim et al., 2019 [34]	RCT	40/44	35/41	64/64	25/25	Pars plana vitrectomy	Sugammadex 2 mg/kg	Neostigmine 1 mg + glycopyrrolate 0.2 mg	Propofol 1.5 mg/kg + remifentanil 0.05-0.1 μg/kg	Desflurane + remifentanil 0.05–0.1 μg/kg/min	Rocuronium 0.6 mg/kg	87/92	PQRS
Muedra et al., 2022 [35]	Cohort	14/7	79/57	74/70	28/29	Aortic valve replacement	Sugammadex 2 mg/kg	Neostigmine 0.03 mg/kg	Propofol 2-2.5 mg/kg + fentanyl 2 mg/kg	Sevoflurane + remifentanil 0.01–0.1 mg/kg/min	Rocuronium 0.8-1.2 mg/kg	273/247	PQRS
Pişkin et al., 2015 [36]	RCT	42/45	71/51	32/37	25/27	Multiple	Sugammadex 2 mg/kg	Neostigmine 0.03 mg/kg + atropine 0.01 mg/kg	Propofol 2 mg/kg + fentanyl 1 μg/kg	Sevoflurane	Rocuronium 0.5 mg/kg	125/124	MMSE/Montreal Cognitive Assessment (MoCA)
Sabuncu et al., 2023 [37]	RCT	41/43	N/A	N/A	47/48	Bariatric	Sugammadex 2 mg/kg	Neostigmine 0.04 mg/kg + atropine 0.02 mg/kg	Propofol 2 mg/kg + fentanyl 1 µg/kg	Desflurane	Rocuronium 0.6 mg/kg	90/91	MMSE
Vu et al., 2023 [38]	Cohort	60/60	31/36	46/46	N/A	Laparoscopic cholecystectomy	Sugammadex 4 mg/kg	Neostigmine 1-2 mg + atropine 0.5-1 mg	Propofol 2 mg/kg + fentanyl 2-3 µg/kg	Sevoflurane + fentanyl 1-2 μg/kg/hour	Rocuronium 0.6 mg/kg	51/67	PQRS

Pooled Analysis

**Figure 2:**
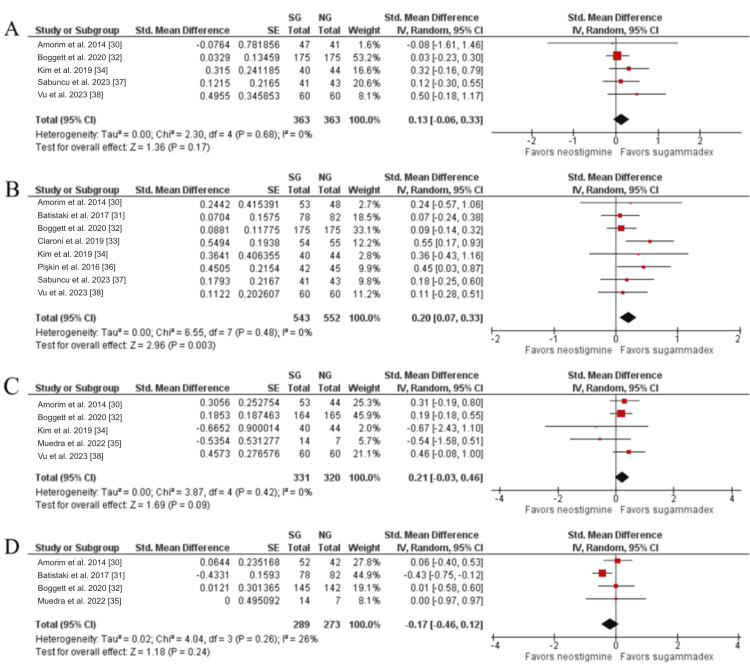
Forest plots of postoperative cognitive recovery A: Immediate [30,32,34,37,38]; B: Early [30-34,36-38]; C: Intermediate [30,32,34,35,38]; D: Late postoperative periods [30-32,35]

No significant differences in cognitive recovery were observed between the sugammadex and neostigmine groups at most postoperative time points: immediate postoperative (≤15 minutes) (SMD 0.13; 95% CI (-0.06, 0.33); p = 0.17; I² = 0%) (Figure 2, panel A); intermediate postoperative (one day after surgery) (SMD 0.21; 95% CI (-0.03, 0.46); p = 0.09; I² = 0%) (Figure 2, panel C); and late postoperative (three to seven days) (SMD -0.17; 95% CI (-0.46, 0.12); p = 0.24; I² = 26%) (Figure 2, panel D). However, early postoperative cognitive recovery (40 to 60 minutes) demonstrated a statistically significant improvement in the sugammadex group compared to the neostigmine group (SMD 0.20; 95% CI (0.07, 0.33); p = 0.003; I² = 0%) (Figure 2, panel B).

Sensitivity Analysis and Quality Assessment

We conducted sensitivity analyses using the leave-one-out method, and the outcome remained statistically significant in all cases. Risk of bias assessment is available in Appendix A. The funnel plot was underpowered due to the small number of studies, and results should be interpreted with caution (Appendix B).

Subgroup Analysis 

In the early postoperative period, subgroup analysis confirmed that the effect remained significant when limited to RCTs and studies using the MMSE, but not with the PQRS cognitive domain. The effect also persisted with neostigmine co-administered with atropine, whereas analyses involving glycopyrrolate, neostigmine alone, or a dose of sugammadex were not feasible due to limited data.

Additional Considerations 

Reports of potential confounding variables varied considerably among studies. Only a few trials provided standardized data on benzodiazepine premedication, intraoperative opioid dosing, or analgesic regimens, precluding formal adjustments or meta-regression. Therefore, although statistical heterogeneity was low, residual confounding from these factors cannot be ruled out.

**Figure 3:**
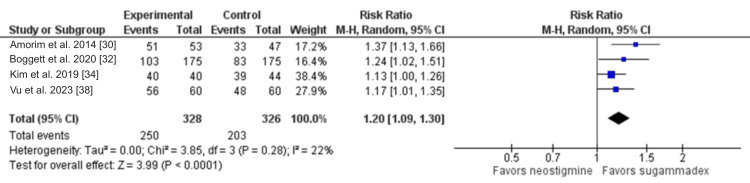
Forest plot of postoperative physiological recovery at 40 minutes Reference numbers of studies showcased in the forest plot: [30,32,34,38]

In the physiological domain, no statistically significant differences were observed at 15 minutes (RR 1.14; 95% CI (0.93, 1.40); p = 0.2; I² = 49%) or day one (RR 0.99; 95% CI (0.96, 1.02); p = 0.5; I² = 0%) (Appendix C). In contrast, a significant improvement in recovery was observed at 40 minutes in the sugammadex group compared to the neostigmine group (RR 1.20; 95% CI (1.09, 1.30); p < 0.0001; I² = 22%) (Figure 3), and significance was maintained in sensitivity analyses.

In the nociceptive domain, no significant differences were found at 15 minutes (RR 1.12; 95% CI (0.98, 1.27); p = 0.09; I² = 0%), 40 minutes (RR 1.06; 95% CI (0.88, 1.27); p = 0.56; I² = 35%), or day one postoperatively (RR 1.00; 95% CI (0.96, 1.05); p = 0.85; I² = 0%) (Appendix D). Similarly, the emotive domain showed no significant differences at any time point: 15 minutes (RR 0.96; 95% CI (0.93, 1.00); p = 0.05; I² = 21%), 40 minutes (RR 0.99; 95% CI (0.95, 1.03); p = 0.49; I² = 31%), or day one postoperatively (RR 1.00; 95% CI (0.97, 1.03); p = 0.83; I² = 0%) (Appendix E). No significant differences were observed in the ADL domain on postoperative day one (RR 1.00; 95% CI [0.87, 1.15]; p = 0.99; I² = 41%) or in overall recovery on postoperative day one (RR 0.95; 95% CI (0.82, 1.09); p = 0.46; I² = 26%) (Appendix F). 

Discussion

In this systematic review and meta-analysis, we evaluated postoperative cognitive recovery and quality of recovery in 1,116 adult patients undergoing elective surgery under general anesthesia, comparing neuromuscular blockade reversal with sugammadex versus neostigmine (± anticholinergics) across nine studies. Sugammadex demonstrated a superior cognitive recovery effect and improved the physiological domain of the PQRS during the early postoperative period (40 to 60 minutes) compared to neostigmine. However, no significant differences were noted at other time points (immediate, intermediate, or late postoperative) or in other recovery domains of the PQRS, such as nociceptive, emotive, activities of daily living, or overall recovery. These findings suggest that sugammadex may offer a short-term advantage over neostigmine in enhancing cognitive and physiological recovery during the early postoperative period, though this benefit is not sustained at later time points or across other recovery domains.

Cognitive recovery in the postoperative period has been evaluated using a variety of scales, yet no consensus exists on the most appropriate tool for assessment [39]. This lack of standardization underscores the challenges in capturing the multifaceted nature of cognitive recovery. In this study, the MMSE and cognitive domains of the PQRS were chosen as measures of cognitive recovery due to their ability to evaluate cognition across multiple domains, including verbal memory, attention, orientation, and executive function [23,40]. These tools are not only practical and straightforward to administer in clinical settings but also hold significant relevance in perioperative management [41,42].

According to Cohen’s criteria [43] the SMD of 0.20 found in our statistical analysis represents a small but not trivial effect size. In the context of cognitive recovery in the early postoperative period, this likely reflects a modest difference between groups, statistically detectable yet likely indicating a subtle rather than substantial improvement in cognitive performance. The analysis showed an I² of 0%, indicating low heterogeneity, and the overall low-to-moderate risk of bias through RoB-2 and ROBINS-1 assessment tools supports the statistical robustness of the pooled results. Even if the effect size were small, the knowledge of the clinical relevance should not be underestimated, since in the early postoperative period, even modest improvements in cognitive and physiological recovery may enhance patient safety and contribute to earlier readiness for discharge from the post-anesthesia care unit (PACU), particularly with vulnerable populations, such as elderly patients or those with preexisting cognitive impairment [44].

This early postoperative benefit of sugammadex may be partially explained by its effects on the cholinergic system, which plays an important role in cognitive functions such as memory, attention, orientation, and motor integration [9,45,46]. In contrast, neostigmine works by inhibiting acetylcholinesterase, which increases acetylcholine levels in the synaptic cleft. However, this mechanism may interfere with the regulation of cholinergic signaling, potentially affecting cognitive processes and the normal transmission of neural stimuli [47]. Additionally, often co-administered anticholinergics, such as atropine and glycopyrrolate, may block central muscarinic receptors, further hindering cognitive pathways and exacerbating potential dysfunction. While these drugs are generally believed to have limited ability to cross the BBB, emerging evidence suggests that anesthesia and perioperative stress may influence the permeability through multiple neuroinflammatory effects [12,13,48,49], which may allow these compounds to impact the central nervous system more significantly than previously thought. However, the clinical relevance of this mechanism remains uncertain.

However, the cognitive improvement observed with sugammadex may not be solely attributed to the cholinergic system. The physiological domain of the PQRS assesses patient vital signs, oxygen support, emergence, and consciousness. The improvement in this domain observed in the sugammadex group during the early postoperative period correlates with the well-established link between optimized hemodynamics and enhanced cognitive function [50-52], potentially explaining, at least in part, the faster cognitive recovery seen in these patients.

The findings of this study align with and expand upon existing literature regarding sugammadex and postoperative recovery. A large multicenter observational study with 29,316 patients undergoing ambulatory surgery compared sugammadex and neostigmine, revealing a small but significant reduction in postoperative length of stay in the ambulatory care unit in the sugammadex group, which translated to lower hospital costs [53]. While the authors attributed this difference to a higher rate of postoperative nausea and vomiting in the neostigmine group, our study, demonstrating faster cognitive and physiological recovery with sugammadex, provides further insight into the reduced PACU time. These findings are further supported by a meta-analysis by Carron et al. [54], who also reported increased PACU length of stay in patients undergoing neuromuscular blockade reversal with neostigmine when compared to sugammadex. This reinforces that, although the high cost of sugammadex limits its widespread use in developing countries, its overall economic impact may be favorable due to reduced hospitalization costs.

The early postoperative improvement in the physiological domain in the sugammadex group also corroborates with current literature. Neostigmine and the often co-administered anticholinergics can negatively impact hemodynamic stability, potentially causing bradycardia, hypotension, or hypertension due to their cholinergic and anticholinergic effects [55]. A prospective randomized study [56] showed more hemodynamic stability in the sugammadex group when compared to neostigmine-atropine in cardiac patients undergoing noncardiac surgery, possibly because of the muscarinic imbalance.

Although POCD and delirium are distinct conditions, they are closely interrelated, and their association is documented in the literature [4]. However, a previous large retrospective cohort study comparing sugammadex and neostigmine found no significant difference in the incidence of postoperative delirium between the two groups [57]. This suggests that the early cognitive improvement observed with sugammadex may not extend to a lower risk of delirium. We were able to demonstrate that sugammadex may lead to a small early improvement of cognitive and physiological recovery, which at last may contribute to the literature evidence of more favorable early postoperative outcomes, such as reduced PACU time and hospital costs.

We acknowledge several limitations in our study. First, the absence of long-term postoperative follow-up prevented us from evaluating persistent cognitive decline and dementia, which are critical outcomes of POCD. Second, the lack of consensus on the optimal scale for assessing cognitive function necessitated the use of standardized mean difference as a measure of association. While this method is well-validated, it limits clinical interpretability. Additionally, the perioperative and intraoperative use of various medications, such as benzodiazepines for premedication, opioids, and volatile anesthetics, can also contribute to cognitive dysfunction. The variability in the administration of those anesthetic protocols across the analyzed studies introduces significant confounding factors, making it challenging to isolate the specific impact of neostigmine on cognitive outcomes, even though those protocols were the same between groups in each study. The heterogeneity of included surgeries was also high, and, as subgroup analysis by type of surgery was not feasible, the real impact of those results in specific surgical settings remains unknown. Moreover, variations in anesthetic techniques, depth of anesthesia monitoring, patient age, and surgical stress across studies may have further contributed to residual confounding. These factors highlight the need for more standardized and long-term studies to better understand the cognitive effects of neuromuscular reversal agents.

## Conclusions

In summary, sugammadex demonstrated a significant improvement in early postoperative cognitive recovery (40 to 60 minutes) compared to neostigmine, while no differences were observed at immediate (≤15 minutes), intermediate (postoperative day one), or late (three to seven days) time points. Additionally, sugammadex showed a significant improvement in physiological recovery of the PQRS at 40 minutes, but no significant differences were found in nociceptive, emotive, ADL, or overall recovery domains at any analyzed time point. 

These findings suggest that the cognitive benefit associated with sugammadex is limited to a short postoperative window and is not sustained over time. Although this early advantage may have potential implications for faster recovery room turnover and reduced length of stay in the PACU, the magnitude of this effect appears small. Importantly, current evidence does not suggest that this transient difference translates into clinically meaningful long-term cognitive outcomes and therefore should be interpreted with caution when considering broader implications for postoperative cognitive function. Notably, the early cognitive benefit remained significant in subgroup analyses restricted to RCTs.

## Figures and Tables

**Figure 4:**
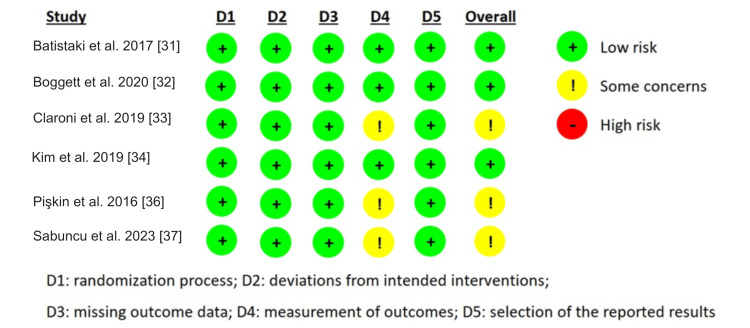
RoB-2 assessment for randomized controlled trials RoB: Risk of bias; Reference numbers of included studies: [31-34,36,37]

**Figure 5:**
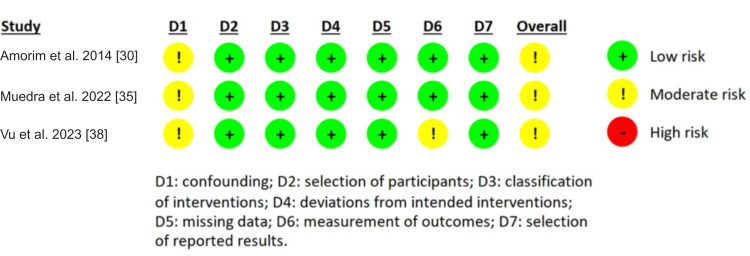
ROBINS-I assessment for observational studies ROBINS-I: Risk of bias in non-randomised studies of interventions; Reference numbers of included studies: [30,35,38]

**Figure 6:**
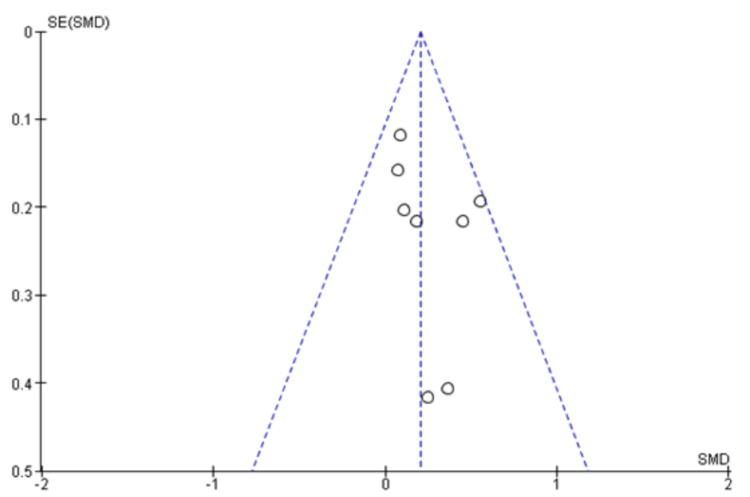
Funnel plot of early cognitive recovery scores from the included studies Reference numbers of included studies: [30-38]

**Figure 7:**
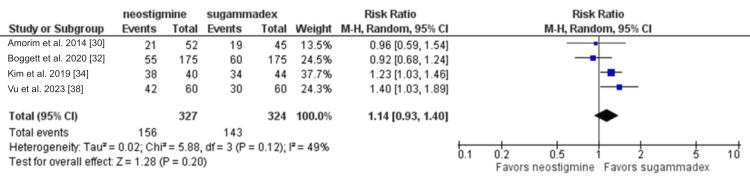
Forest plot of physiological recovery at 15 minutes M-H: Mantel-Haenszel, Reference numbers of included studies: [30,32,34,38]

**Figure 8:**
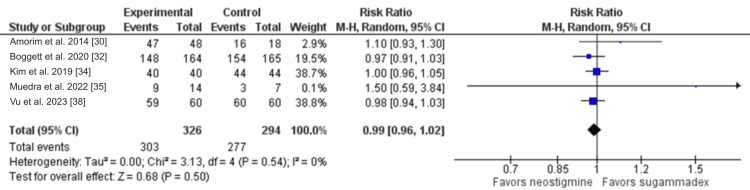
Forest plot of physiological recovery on postoperative day one M-H: Mantel-Haenszel, Reference numbers of included studies: [30,32,34,35,38]

**Figure 9:**
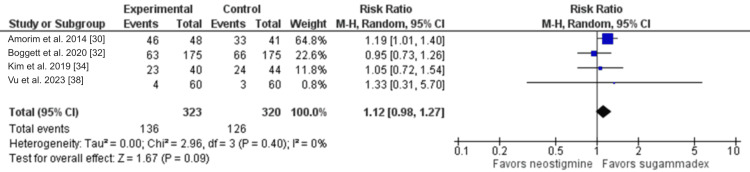
Forest plot of nociceptive recovery at 15 minutes M-H: Mantel-Haenszel, Reference numbers of included studies: [30,32,34,38]

**Figure 10:**
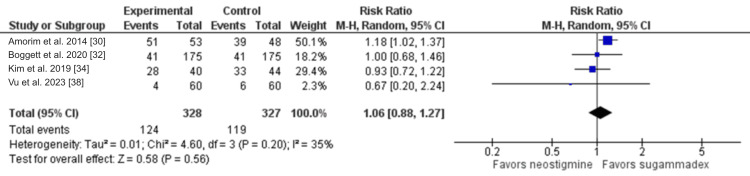
Forest plot of nociceptive recovery at 40 minutes M-H: Mantel-Haenszel, Reference numbers of included studies: [30,32,34,38]

**Figure 11:**
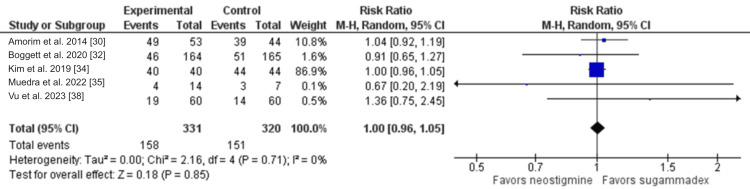
Forest plot of nociceptive recovery on postoperative day one M-H: Mantel-Haenszel, Reference numbers of included studies: [30,32,34,35,38]

**Figure 12:**
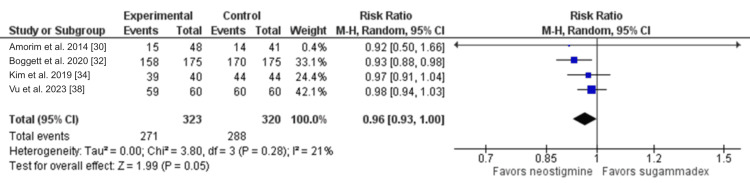
Forest plot of emotive recovery at 15 minutes M-H: Mantel-Haenszel, Reference numbers of included studies: [30,32,34,38]

**Figure 13:**
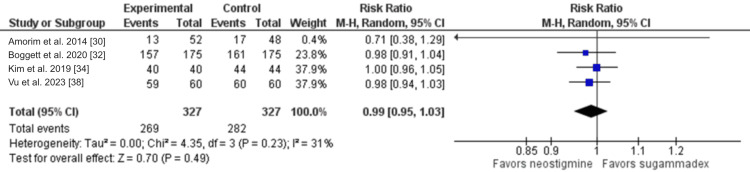
Forest plot of emotive recovery at 40 minutes M-H: Mantel-Haenszel, Reference numbers of included studies: [30,32,34,38]

**Figure 14:**
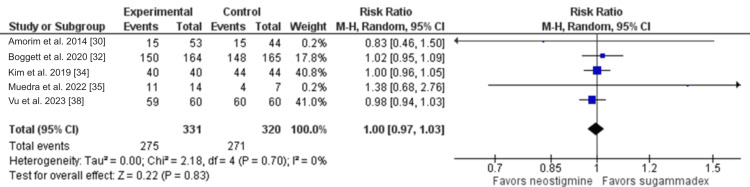
Forest plot of emotive recovery on postoperative day one M-H: Mantel-Haenszel, Reference numbers of included studies: [30,32,34,35,38]

**Figure 15:**
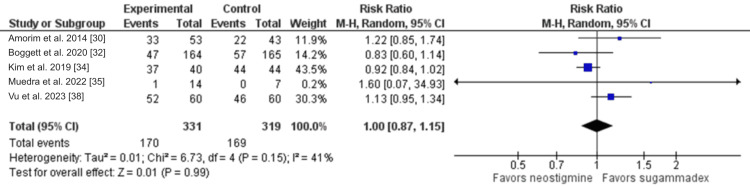
Forest plot of ADL recovery on postoperative day one ADL: Activities of daily living, M-H: Mantel-Haenszel, Reference numbers of included studies: [30,32,34,35,38]

**Figure 16:**
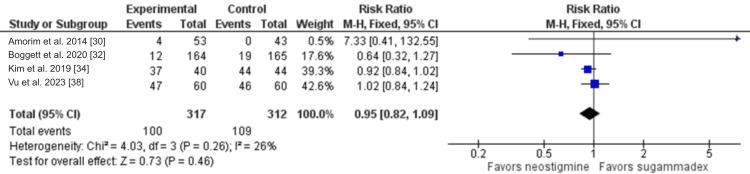
Forest plot of overall recovery on postoperative day one M-H: Mantel-Haenszel, Reference numbers of included studies: [30,32,34,38]

## Appendices

Appendix A 

Figures 4-5 feature the risk of bias assessment.

Appendix B 

Figure 6 showcases the funnel plot, which was underpowered due to the small number of studies. Therefore, results should be interpreted with caution.

Appendix C 

Figures 7-8, featured below, show the forest plots of postoperative physiological recovery at 40 minutes and on day 1 postoperatively.

Appendix D

Figures 9-11 feature the forest plots of nociceptive recovery at 15 minutes, 40 minutes, and on postoperative day one.

Appendix E

Figures 12-14 feature the forest plots of emotive recovery at 15 minutes, 40 minutes, and on day one postoperatively.

Appendix F 

Figure 15 features the forest plot for the ADL domain on postoperative day one, and Figure 16 shows the forest plot for overall recovery on postoperative day one.
